# Short-term effects of ambient ozone on pediatric pneumonia hospital admissions: a multi-city case-crossover study in China

**DOI:** 10.1265/ehpm.25-00242

**Published:** 2025-09-23

**Authors:** Huan Wang, Huan-Ling Zeng, Guo-Xing Li, Shuang Zhou, Jin-Lang Lyu, Qin Li, Guo-Shuang Feng, Hai-Jun Wang

**Affiliations:** 1 Department of Maternal and Child Health, School of Public Health, Peking University, National Health Commission Key Laboratory of Reproductive Health, Beijing, China; 2 Department of Oncology, Xiaolan People’s Hospital of Zhongshan, the Fifth People’s Hospital of Zhongshan, Zhongshan, China; 3 Department of Occupational and Environmental Health Sciences, School of Public Health, Peking University, Beijing, China; 4 Environmental Research Group, MRC Centre for Environment and Health, Imperial College London, London, UK; 5 Climate, Air Quality Research Unit, School of Public Health and Preventive Medicine, Monash University, Melbourne, Australia; 6 Big Data Center, Beijing Children’s Hospital, Capital Medical University, National Center for Children’s Health, Beijing, China

**Keywords:** Ozone, Pediatric pneumonia, Case-crossover study

## Abstract

**Background:**

Children’s respiratory health demonstrates particular sensitivity to air pollution. Existing evidence investigating the association between short-term ozone (O_3_) exposure and childhood pneumonia remains insufficient and inconsistent, especially in low- and middle-income countries (LMICs).

**Method:**

To provide more reliable and persuasive evidence, we implemented a multi-city, time-stratified case-crossover design with a large sample size, using data from seven representative children’s hospitals across major geographical regions in China. To avoid the impact of the COVID-19 pandemic, individual-level medical records of inpatient children under 6 years of age diagnosed with pneumonia during 2016–2019 were collected. Conditional logistic regression models were fitted for each city, and city-specific estimates were pooled through a meta-analysis using a random-effects model.

**Results:**

In total, the study included 137,470 pediatric pneumonia hospital admissions. The highest pooled estimate for O_3_ occurred at lag0–1, with a 10 µg/m^3^ increase in O_3_ associated with a 1.57% (95% CI: 0.67%–2.48%) higher risk of pediatric pneumonia hospital admissions. Stratified analyses indicated that the effects of O_3_ were robust across different sexes, age groups, and admission seasons. We also observed a statistically significant increase in risk associated with O_3_ concentrations exceeding the World Health Organization Air Quality Guidelines (WHO-AQGs).

**Conclusions:**

This study revealed a significant positive association between O_3_ and pediatric pneumonia hospital admissions. Our findings substantially strengthen the evidence base for the adverse health impacts of O_3_, underscoring the importance of O_3_ pollution control and management in reducing the public health burden of pediatric pneumonia.

**Supplementary information:**

The online version contains supplementary material available at https://doi.org/10.1265/ehpm.25-00242.

## Graphical Abstract

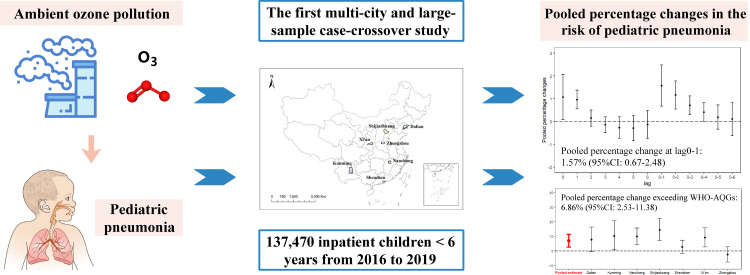

## 1. Introduction

Pneumonia is the leading cause of global mortality and morbidity in children [1], accounting for 18.7% of child deaths under 5 years of age worldwide in 2019 [2]. Therefore, identifying the risk factors of pediatric pneumonia is crucial for reducing the disease burden in children. Given that the respiratory system is the most directly affected organ by air pollutants, considerable progress has been made in understanding the effects of air pollution on pneumonia. Children face greater risks than adults from the adverse health effects of air pollution due to a combination of behavioral, environmental, and physiological factors. These include a higher resting metabolic rate, a larger lung surface area relative to body weight, an immature lung and immune system, and more time spent outdoors [3–5]. It is essential to investigate the association between air pollution and pediatric pneumonia, especially in low- and middle-income countries (LMICs), where severe ambient air pollution is common.

To date, compared with fine particulate matter (PM_2.5_), far less attention has been paid to the association between ambient ozone (O_3_) exposure and pneumonia in children. One meta-analysis of 17 studies from 8 countries showed a positive linear association between short-term daily O_3_ levels and pediatric hospital admissions due to pneumonia [6], but it included only one study with a very small sample size from China. In fact, O_3_ is now one of the predominant air pollutants and represents an increasing public health challenge in China. According to the China Ecology and Environment Yearbook, the annual average concentration of O_3_ was 144 µg/m^3^ in 2023, well above the 100 µg/m^3^ recommended by the World Health Organization Air Quality Guidelines (WHO-AQGs), which was just similar to the period before the COVID-19 pandemic. Due to China’s vast territory and geographic diversity, findings on the association between O_3_ and pediatric pneumonia may have important implications for other LMICs.

Although a growing body of literature has explored the effects of O_3_ exposure on pediatric pneumonia, existing evidence remains insufficient and inconsistent. Previous studies have mostly relied on data from a single hospital or city with small sample sizes, limiting the statistical power to provide reliable conclusions [7–9]. To our knowledge, only two multi-city studies have been conducted in China. Using time-series analysis, one study across four cities (Guangzhou, Shanghai, Wuhan, and Xining) found no association between O_3_ and outpatient visits for pediatric respiratory diseases [10], whereas another study in 25 cities reported that O_3_ exposure was associated with increased hospitalizations for childhood pneumonia [11]. This inconsistency may stem from the inherent ecological bias of time-series studies, which use aggregated daily counts and cannot control for individual-level confounders [12]. By contrast, the case-crossover design allows researchers to control the impacts of these confounders more effectively by comparing each subject’s exposure to their own baseline exposure at different time points [13]. Therefore, we argue that a multi-city case-crossover study, incorporating geographically diverse regions and a large sample size, is essential to comprehensively quantify the short-term effects of O_3_ exposure on pediatric pneumonia hospital admissions.

In this study, we analyzed data on pediatric pneumonia hospital admissions from seven large children’s hospitals, each located in a representative geographical region of China, during 2016–2019 to avoid the impact of the COVID-19 pandemic. We applied a two-stage, time-stratified case-crossover design to systematically assess the short-term effects of ambient O_3_ on pediatric pneumonia hospital admissions. Potential effect modifiers—including sex, age, and season of hospital admission—were further explored to enhance understanding of these associations. Finally, we examined whether O_3_ levels exceeding the WHO-AQGs were associated with significantly increased risk of pediatric pneumonia.

## 2. Methods

### 2.1 Study areas

**Fig. 1:**
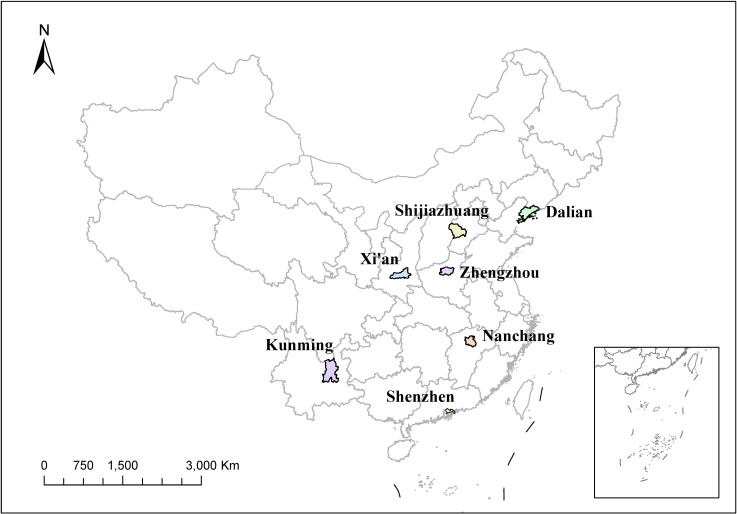
Locations of the seven cities included in the study

Based on natural environmental variation, China is conventionally divided into seven major geographical regions: Northeastern, North, East, South, Central, Northwestern, and Southwestern China. To ensure diverse geographic coverage, one representative city was selected from each region—Dalian, Shijiazhuang, Nanchang, Shenzhen, Zhengzhou, Xi’an, and Kunming. The locations of the selected cities are shown in Fig. 1, spanning a total land area of over 76,000 km^2^.

Climatic heterogeneity is evident across these cities. Dalian exhibits a temperate monsoon climate with maritime influence, characterized by mild summers and cold, dry winters. Nanchang has a subtropical monsoon climate with extreme summer heat and high humidity. Xi’an experiences a warm-temperate semi-humid climate with pronounced continental diurnal temperature variation. Shijiazhuang is representative of a dry continental monsoon climate, with frequent spring dust storms and concentrated summer rainfall. Shenzhen has a humid subtropical marine climate, dominated by typhoons and year-round warmth. Kunming, also known as the “Spring City,” features a subtropical highland climate with minimal annual temperature fluctuation. Zhengzhou displays transitional warm-temperate traits, experiencing both extreme summer droughts and heavy rain events.

We collected electronic medical records from a large children’s hospital in each city through the FUTang Updating medical REcords (FUTURE) database, the first non-profit social service organization in China dedicated to pediatric medical research. The FUTURE database follows rigorous data quality control protocols; detailed documentation is available elsewhere [14]. The Ethics Committee of Beijing Children’s Hospital reviewed and approved the study protocol prior to data retrieval (2020-k-10).

### 2.2 Hospital admissions

At seven children’s hospitals, individual-level medical records of inpatient children under 6 years of age were extracted for the period from January 1, 2016, to December 31, 2019 to avoid the impact of the COVID-19 pandemic. Extracted variables included primary diagnosis, date of hospitalization, residential address, sex, and age. To avoid exposure misclassification and confounding bias due to readmissions, we excluded patients who were not residents of the same city (based on residential address) and repeat admissions for the same individual. According to the 10th edition of the International Classification of Diseases (ICD-10) and the pediatric pneumonia definition from the National Health Commission of China’s single-disease management guidelines (www.nhc.gov.cn), pediatric pneumonia hospital admissions were identified by ICD-10 codes J13–J16 and J18. Specifically, J13–J16 denote pneumonia classified by etiology, while J18 indicates pneumonia classified by anatomical location.

### 2.3 Air pollutants and meteorological conditions

Daily average concentrations of O_3_, PM_2.5_ and other three gaseous pollutants (SO_2_, NO_2_ and CO) were calculated at the city level by averaging all raster data within each city’s boundary, based on the China High Air Pollutants (CHAP) dataset. This dataset provides spatial resolutions of 1 km × 1 km for PM_2.5_ and 10 km × 10 km for O_3_, SO_2_, NO_2_ and CO (https://weijing-rs.github.io/product.htm). The CHAP dataset is widely recognized as a credible and robust source for air pollution exposure assessment in China and has been extensively used in epidemiological research [15, 16]. Meteorological data, including daily mean air temperature and relative humidity, were obtained from the Resource and Environmental Science Data Platform (www.resdc.cn), managed by the Institute of Geographic Sciences and Natural Resources Research, Chinese Academy of Sciences. As with air pollution measures, daily meteorological conditions were estimated using the average of all monitoring stations in each city.

### 2.4 Statistical analyses

Following a time-stratified case-crossover study design [13], we employed a two-stage approach to assess the association between short-term ambient O_3_ exposure and pediatric pneumonia hospital admissions.

In the first stage, we evaluated the short-term effects of ambient O_3_ pollution by conducting time-stratified case-crossover analyses separately for each city. For each pediatric pneumonia case, the case day was defined as the hospital admission date with a primary diagnosis of pneumonia. Corresponding control days were selected as the same day of the week within the same month and year, to control for long-term and seasonal trends while adjusting for individual-level confounders that remain constant over short time intervals—such as age, sex, race, education, body weight, lifestyle, and socioeconomic status [10, 17, 18]. Each case thus had three or four control days. Conditional logistic regression models were fitted for each city, using daily average O_3_ concentrations as the independent variable, while adjusting for daily PM_2.5_ concentrations, meteorological factors (daily mean temperature and relative humidity), and public holidays. To account for lagged exposure effects, we examined both single-day lags (lag0 to lag6) and multi-day moving averages (lag0–1 to lag0–6). The model was specified as follows:

$$\begin{array}{rl} & \text{Logit }P\;(\text{case = 1 in stratum }i)\\ & \;=\alpha_{\text{stratum i}}+\beta \;({\text{O}}_{3},\;{\text{PM}}_{2.5})+\mathrm{ns}\;(\text{temperature},\;\mathrm{df}=3)\\ & \;+\mathrm{ns}\;(\text{relative humidity},\;\mathrm{df}=3)+\text{holiday} \end{array}$$ (1)

where *P* is the conditional probability of being a case in stratum *i*, defined by matched case and control days for the same individual. The coefficient β represents the change in odds of pediatric pneumonia admissions associated with a 10 µg/m^3^ increase in O_3_, adjusted for PM_2.5_. The terms *ns*() denote natural cubic spline functions, with 3 degrees of freedom (df) for both temperature and relative humidity, based on prior literature [6]. Holiday is a binary variable indicating whether the date is a national public holiday as defined by the Chinese government.

In the second stage, we pooled city-specific estimates using meta-analysis with a random-effects model, consistent with previous studies. We then converted odds ratios (ORs) to percentage changes in the risk of pediatric pneumonia hospital admissions per 10 µg/m^3^ increase in O_3_ exposure for each lag effect [19]. Percentage changes were calculated as (OR − 1) × 100.

The lag pattern with the largest effect estimate was selected for stratified analyses by sex (male and female), age group (≤3 years and 3–6 years), and season of hospital admission (warm season: April to September; cold season: October to March), in accordance with prior research [20]. We used the Z-test to determine whether differences in effect estimates between subgroups were statistically significant, based on the formula: $({\text{E}}_{1}-{\text{E}}_{2})\;\pm \sqrt{{{\text{SE}}_{1}}^{2}+{{\text{SE}}_{2}}^{2}}$ , where E_1_ and E_2_ are the effect estimates for each subgroup, and SE_1_ as well as SE_2_ are their corresponding standard errors. We also evaluated the number of excess admissions attributable to O_3_ concentrations exceeding WHO-AQGs, using exposures below 100 µg/m^3^ as the reference level.

Finally, we conducted sensitivity analyses to assess the robustness of our main findings. First, we applied 2 and 4 df instead of 3 df for daily mean air temperature and relative humidity in the main model, to examine the potential confounding effects of alternative model specifications. Second, because approximately 50% of hospital admissions in the FUTURE database included detailed residential addresses (i.e., addresses could be pinpointed to specific neighborhoods) while the remaining cases were only geolocatable to the city level, we improved exposure assessment by restricting analysis to this subgroup. Specifically, we assigned the raster air pollution and meteorological data from the nearest monitoring station to individuals with available address information. Third, we additionally adjusted the other three gaseous pollutants (SO_2_, NO_2_ and CO) by separately including each pollutant on both case and control days. All analyses were performed using Python version 3.10 and R version 4.2.0. A two-tailed *P* value < 0.05 was considered statistically significant.

## 3. Results

### 3.1 Characteristics of the study population, air pollutants, and meteorological variables

A total of 137,470 pneumonia hospital admissions among children under 6 years of age from seven representative cities between January 1, 2016, and December 31, 2019, were included in this study. The demographic characteristics of the study population are presented in Table S1.

Boys and children under 3 years old accounted for 60.6% and 71.4% of all cases, respectively. The proportion of pediatric pneumonia admissions during the warm season was higher than during the cold season (59.7% vs. 40.3%). Table 1 presents summary statistics for air pollutant concentrations and meteorological conditions on case days. The median daily concentrations were 83.9 µg/m^3^ for O_3_ (interquartile range: 58.0–118.9 µg/m^3^), 39.5 µg/m^3^ for PM_2.5_ (interquartile range: 26.3–62.9 µg/m^3^), 14.9 °C for temperature (interquartile range: 6.0–23.0 °C), and 68.0% for relative humidity (interquartile range: 52.5–80.1%). Table S2 shows O_3_ concentrations on case days across warm and cold seasons. Table S3 presents Spearman’s correlation coefficients among air pollutants and meteorological variables on case days, indicating that O_3_ was generally positively correlated with temperature, while the relationship between O_3_ and PM_2.5_ varied across cities.

**Table 1:** Summary distribution of air pollutant concentrations and meteorological conditions on case days

**City**	**Median (25th, 75th)**			

	**O_3_ (µg/m^3^)**	**PM_2.5_ (µg/m^3^)**	**Temperature (°C)**	**Relative humidity (%)**
Dalian	85.4 (62.0, 113.1)	33.4 (23.8, 48.4)	9.8 (0.2, 19.3)	60.6 (47.1, 77.5)
Kunming	84.4 (71.0, 102.6)	21.6 (17.2, 27.3)	15.9 (11.9, 19.8)	71.3 (60.2, 79.8)
Nanchang	83.6 (56.7, 110.5)	39.1 (26.7, 56.7)	13.2 (7.8, 22.7)	77.2 (67.2, 86.8)
Shijiazhuang	82.7 (48.4, 133.4)	58.2 (39.7, 91.3)	9.8 (0.9, 22.6)	55.2 (41.4, 71.1)
Shenzhen	87.7 (62.6, 122.3)	26.1 (18.1, 35.8)	25.0 (20.1, 28.2)	80.0 (72.0, 86.0)
Xi’an	78.2 (55.8, 111.2)	42.2 (29.2, 66.7)	11.5 (4.4, 20.1)	67.0 (54.7, 78.8)
Zhengzhou	86.7 (56.0, 138.0)	55.2 (36.9, 87.1)	12.3 (4.5, 22.0)	58.7 (44.6, 73.4)
Overall	83.9 (58.0, 118.9)	39.5 (26.3, 62.9)	14.9 (6.0, 23.0)	68.0 (52.5, 80.1)

Abbreviations: 25th, the 25th percentile; 75th, the 75th percentile

### 3.2 Association between short-term ambient O_3_ exposure and pediatric pneumonia hospital admissions

**Fig. 2:**
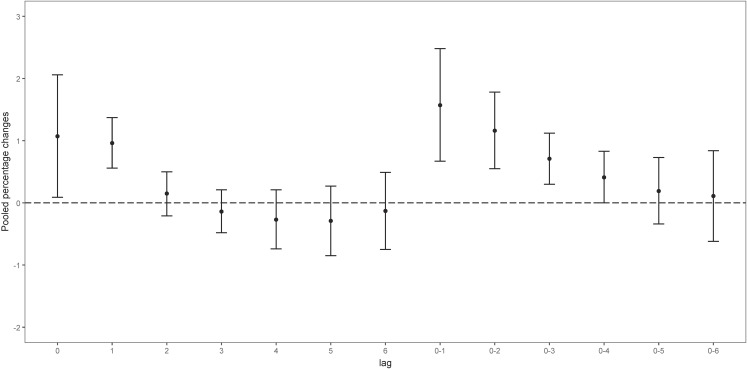
Pooled percentage change in the risk of pediatric pneumonia per 10 µg/m^3^ O_3_ increase Abbreviations: lag1, the previous day; lag2, the previous 2 days; lag3, the previous 3 days; lag4, the previous 4 days; lag5, the previous 5 days; lag6, the previous 6 days; lag0–1, moving average of the case day and the previous day; lag0–2, moving average of the case day and the previous 2 days; lag0–3, moving average of the case day and the previous 3 days; lag0–4, moving average of the case day and the previous 4 days; lag0–5, moving average of the case day and the previous 5 days; lag0–6, moving averages of the case day and 1 to 6 preceding days.

Using a two-stage time-stratified case-crossover approach, we found statistically significant associations between short-term ambient O_3_ exposure and pediatric pneumonia hospital admissions. Table S4 and Fig. 2 show the pooled percentage changes in pneumonia admission risk per 10 µg/m^3^ increase in O_3_ exposure across different lag periods. Overall, short-term ambient O_3_ exposure was significantly associated with increased risk of pediatric pneumonia hospitalization at lag0, lag1, lag0–1, lag0–2, and lag0–3. The pooled effect estimates declined with increasing lag duration, with the strongest association observed at lag0–1. Specifically, a 10 µg/m^3^ increase in O_3_ at lag0–1 was associated with a 1.57% (95% confidence interval [CI]: 0.67%–2.48%) increase in the risk of pneumonia hospital admissions in children. City-specific estimates of risk change are presented in Table S5. City-specific exposure–response curves appeared generally linear (Fig. S1).

### 3.3 Stratified analyses by sex, age, and admission season

Stratified analyses showed that pooled effect estimates of O_3_ at lag0–1 were slightly higher among girls, children aged 3–6 years, and during the warm season. However, no statistically significant differences were observed between subgroups, indicating that the association between O_3_ exposure and pediatric pneumonia hospital admissions was not significantly modified by sex, age group, or season of admission (Table 2; Table S6).

**Table 2:** Percentage change in the risk per 10 µg/m^3^ O_3_ increase in stratified analyses (lag0–1)

**Subgroup**	**Percentage Change**	**95% CI**	***P* value**	***P* for interaction**
**Sex**				0.398
Male	1.53	(0.61, 2.46)	**0.001**	
Female	1.59	(0.45, 2.75)	**0.006**	

**Age group**				0.317
≤3 y	1.46	(0.54, 2.39)	**0.002**	
3–6 y	1.97	(0.68, 3.27)	**0.003**	

**Admission season**				0.331
Warm	1.35	(0.13, 2.59)	**0.030**	
Cold	0.90	(0.08, 1.72)	**0.032**	

Abbreviations: CI, Confidence Interval; lag0–1, moving average of the case day and the day before.

### 3.4 Excess admissions analyses

Table 3 shows that O_3_ exposure levels exceeding WHO-AQGs (100 µg/m^3^) were associated with significant increases in pediatric pneumonia hospital admissions. Notably, the overall percentage change in risk was 6.86% (95% CI: 2.53%–11.38%), suggesting that stricter O_3_ control measures aligned with WHO-AQGs may be warranted to protect children’s respiratory health.

**Table 3:** Percentage change in the risk associated with O_3_ > WHO-AQGs (100 µg/m^3^) at lag0–1

**City**	**Percentage Change**	**95% CI**	***P* value**
Dalian	7.68	(−0.37, 16.38)	0.062
Kunming	10.26	(0.76, 20.65)	**<0.05**
Nanchang	9.89	(4.40, 15.68)	**<0.001**
Shijiazhuang	14.45	(6.99, 22.43)	**<0.001**
Shenzhen	2.75	(−1.64, 7.34)	**<0.05**
Xi’an	9.11	(2.74, 15.88)	0.224
Zhengzhou	−2.53	(−7.66, 2.88)	0.352
Pooled estimate	6.86	(2.53, 11.38)	**<0.05**

Reference group: O_3_ concentrations below 100 µg/m^3^ at lag0–1

Abbreviations: CI, Confidence Interval; lag0–1, moving average of the case day and the previous day

### 3.5 Sensitivity analyses

Compared with the original model, variations in degrees of freedom for daily mean air temperature and relative humidity, along with improved exposure matching for air pollution and meteorological data, did not materially alter the observed association between O_3_ exposure and pediatric pneumonia hospital admissions (Table 4 and Table S7). Additionally, in sensitivity analysis models that incorporated one of the three other criteria gaseous pollutants (SO_2_, NO_2_, or CO), the pooled effect estimates for O_3_ at lag0–1 remained consistent (Table S8).

**Table 4:** Percentage change in the risk per 10 µg/m^3^ O_3_ increase in sensitivity analyses (lag0–1)

**Model**	**Percentage Change**	**95% CI**	***P* value**	***P* for interaction**
Original model	1.57	(0.67, 2.48)	**<0.001**	reference
Model 1	1.52	(0.73, 2.32)	**<0.001**	0.399
Model 2	1.58	(0.69, 2.47)	**<0.001**	0.398
Model 3	1.45	(1.01, 1.90)	**<0.001**	0.388

Model 1: Meteorological conditions modeled using 2 degrees of freedom in the natural cubic spline function

Model 2: Meteorological conditions modeled using 4 degrees of freedom in the natural cubic spline function

Model 3: Subgroup of subjects (*n* = 64,525) with residential address data matched to raster air pollutant data and nearest meteorological station

Abbreviations: CI, Confidence Interval; lag0–1, moving average of the case day and the day before.

## 4. Discussion

Using a two-stage time-stratified case-crossover design, our findings revealed a significant positive association between short-term ambient O_3_ exposure and pediatric pneumonia hospital admissions. Stratified analyses indicated that this association was not significantly modified by sex, age group, or season of admission. We also found a substantial increase in childhood pneumonia hospitalizations associated with O_3_ exposures exceeding the WHO-AQGs. To our knowledge, this is the first multi-city, large-sample case-crossover study to examine the short-term effects of ambient O_3_ pollution on pediatric pneumonia hospital admissions, providing critical and novel epidemiological evidence for assessing the disease burden of childhood pneumonia attributable to O_3_ exposure.

In this study, short-term ambient O_3_ exposure was significantly associated with an increased risk of pediatric pneumonia hospitalizations at lag0, lag1, lag0–1, lag0–2, and lag0–3. The strongest effect was observed at lag0–1, where a 10 µg/m^3^ increase in O_3_ was associated with a 1.57% (95% CI: 0.67%–2.48%) increase in the risk of pneumonia hospitalization in children. Previous multi-city studies examining O_3_ exposure and childhood pneumonia risk remain limited. A time-series study conducted in 25 Chinese cities reported a 0.20% increase in pneumonia hospitalizations per 10 µg/m^3^ increase in O_3_ at lag0–1 [11], consistent with findings from a nationwide time-series study in South Korea [12] and a U.S. study on emergency department visits for respiratory illness in 17 states [21]. However, time-series designs rely on aggregated daily admission counts and are susceptible to ecological bias, which may reduce their sensitivity to detect individual-level associations. In contrast, the self-matching feature of the case-crossover design inherently controls for time-invariant individual-level confounders—such as demographic characteristics, behaviors, and socioeconomic status—thereby improving causal inference. A time-stratified case-crossover study conducted in 16 cities in California also found that short-term O_3_ exposure was positively associated with pediatric emergency department visits for pneumonia among children aged 0–4 years [22]. However, the applicability of those findings to China is limited due to substantial cross-country differences in ambient O_3_ concentrations. Additionally, a meta-analysis of 33 Chinese studies—spanning both time-series and case-crossover designs—reported a 0.74% (95% CI: 0.01%–1.46%) increase in outpatient visits for pediatric respiratory diseases per 10 µg/m^3^ increase in O_3_ [23]. It is important to note that pediatric respiratory diseases encompass a broad spectrum, including upper respiratory infections and non-infectious respiratory conditions. As a result, studies aggregating across this category may underestimate the true burden of O_3_-specific pneumonia. Therefore, our study provides new and reliable evidence supporting a specific link between short-term ambient O_3_ exposure and pediatric pneumonia hospital admissions—findings that are essential for informing public health efforts to mitigate childhood respiratory disease burden.

At the single-city level in this study, we observed significant positive associations between O_3_ exposure and pediatric pneumonia hospital admissions in six cities (Dalian, Shijiazhuang, Nanchang, Shenzhen, Zhengzhou, and Xi’an), with varying magnitudes of effect estimates. In contrast, one city—Kunming—showed no significant association across any of the selected lag patterns. Additionally, a negative effect estimate for O_3_ was found in Zhengzhou, where the concentration exceeded WHO-AQGs (100 µg/m^3^) at lag0–1, although it was not statistically significant. These findings highlight potential heterogeneity in the O_3_–pneumonia relationship across geographic locations. For example, a case-crossover study in Brisbane, Australia, found that O_3_ exposure was associated with increased emergency department visits for pneumonia in children. This aligns with single-city studies conducted in Vietnam [24] and Turkey [25]. Conversely, a time-series study involving four Chinese cities [10] and several other single-city studies in China [26, 27] found no significant association between O_3_ exposure and pediatric respiratory hospital admissions. The observed heterogeneity in effect estimates may be attributed to differences in ambient O_3_ levels, population vulnerability, and healthcare-seeking behaviors—such as the “harvesting effect,” whereby in high-level air pollution cities children were taken to hospitals even for mild symptoms, potentially masking the full effect of peak O_3_ exposure [28]. This geographic variation further underscores the importance of including regionally diverse populations in study designs to capture the full spectrum of O_3_-related health effects in children.

The biological mechanisms underlying the association between O_3_ exposure and pneumonia risk in children may involve several interrelated pathways. First, as a potent oxidant, inhaled O_3_ rapidly generates reactive oxygen and nitrogen species in the respiratory tract, which compromise alveolar epithelial integrity and amplify pulmonary inflammation through oxidative stress cascades [29–31]. Second, emerging evidence suggests that O_3_ can activate sensory neurons, triggering autonomic nervous system imbalances that increase airway hyperresponsiveness and infection risk [32]. Additionally, O_3_ may disrupt immune homeostasis by altering immune cell trafficking and function, thereby creating an immunosuppressive environment conducive to infection [33, 34]. Recent panel studies have also shown that O_3_ activates the hypoxia-inducible factor-1α pathway, reducing peripheral oxygen saturation and impairing mucociliary clearance, thus exacerbating pulmonary injury [35]. Given children’s heightened vulnerability to air pollution—due to physiological immaturity, behavioral factors (e.g., longer outdoor exposure), and environmental conditions—they constitute a critical population for targeted air quality interventions to mitigate the adverse health effects of O_3_.

This study revealed comparable effect estimates of O_3_ exposure across stratified subgroups, including sex, age, and season of admission. To date, the evidence for effect modification by individual characteristics remains inconclusive. For example, a case-crossover study conducted in southern China found no notable modification by age or sex in the association between ambient O_3_ exposure and emergency department visits among children [36]. In contrast, another case-crossover study in Australian children reported sex- and age-specific differences in the health effects of air pollution [37]. Additionally, most previous studies reported greater associations between O_3_ exposure and pneumonia in the warm season than in the cool season [22, 38, 39]. This observed association may be attributed to enhanced photochemical reaction rates promoting O_3_ formation through more efficient precursor conversion, coupled with increased pediatric outdoor exposure during warmer seasons. However, a study in two East Asian cities (Hong Kong and Taipei) observed stronger effects of O_3_ on pneumonia hospital admissions during the cool season compared to the warm season [28], consistent with a nationwide time-series study in South Korea [12]. The underlying reasons for these inconsistencies remain unclear but may involve differences in developmental status, physiological susceptibility, behavioral factors, and individual-level exposure patterns.

Compared with prior research, this study has several notable strengths. First, it is the first multi-city case-crossover study with a large sample size to examine the short-term effects of ambient O_3_ on the risk of pneumonia in children. Our findings provide robust epidemiological evidence with strong statistical power and broad relevance, offering important implications for pediatric health in diverse settings. Second, the self-matching feature of the case-crossover design offers more effective control for time-invariant individual confounders than conventional time-series models, thereby enhancing the internal validity of our results. Third, our findings confirm an overall significant increase in pediatric pneumonia hospitalizations associated with O_3_ levels exceeding WHO-AQGs, reinforcing the need for stricter regulatory policies aligned with WHO guidelines to protect child health.

This study has several limitations. First, because the data on pediatric pneumonia hospital admissions were derived from seven representative cities in China, the generalizability of the findings to smaller cities and rural areas may be limited. Second, potential exposure misclassification may have occurred due to the use of daily average concentrations of air pollutants and meteorological conditions at the city level. However, previous studies have considered city-level exposure assessment acceptable for estimating short-term air pollution effects [40]. This approach is further supported by the consistency of our sensitivity analyses, which used individual-level exposure assessments based on high-resolution raster data for air pollutants. Third, due to the low proportion of etiologically diagnosed pediatric pneumonia cases in our study, we were unable to perform further subgroup analyses by pathogen type, which highlights the need for future studies to enable pathogen-specific analyses.

## 5. Conclusion

This multi-city case-crossover study demonstrated that short-term ambient O_3_ exposure is associated with an increased risk of pediatric pneumonia hospital admissions. The strongest pooled effect was observed at lag0–1, and the association remained robust across different sexes, age groups, and seasons of admission. These findings provide new and reliable scientific evidence on the impact of O_3_ exposure on pediatric pneumonia, underscoring the need for strengthened air quality management to prevent and control childhood pneumonia. In addition, this study offers a strong foundation for policymakers to design strategies aimed at reducing the public health burden of pediatric pneumonia attributable to ambient O_3_.

## Abbreviations

O_3_: ozone
LMICs: low- and middle-income countries
WHO-AQGs: World Health Organization Air Quality Guidelines
PM_2.5_: fine particulate matter
FUTURE database: the FUTang Updating medical REcords database
ICD-10: the 10th edition of the International Classification of Diseases
CHAP dataset: the China High Air Pollutants dataset

## Supplementary information

**Additional file 1:** **Table S1.** Demographic characteristics of the study population. **Table S2.** Summary distribution of O_3_ concentrations on case days across warm and cold seasons. **Table S3.** Spearman’s correlation coefficients among air pollutants and meteorological conditions on case days. **Table S4.** Pooled percentage change in the risk of pediatric pneumonia per 10 µg/m^3^ O_3_ increase. **Table S5.** City-specific percentage change in the risk of pediatric pneumonia hospital admissions per 10 µg/m^3^ increase in O_3_ exposure at different lag periods. **Table S6.** City-specific and pooled percentage change in the risk of pediatric pneumonia hospital admissions per 10 µg/m^3^ increase in O_3_ exposure at lag0–1 in stratified analyses. **Table S7.** City-specific and pooled percentage change in the risk of pediatric pneumonia hospital admissions per 10 µg/m^3^ increase in O_3_ exposure at lag0–1 in sensitivity analyses. **Table S8.** City-specific and pooled percentage change in the risk of pediatric pneumonia hospital admissions per 10 µg/m^3^ increase in O_3_ exposure at lag0–1 with additional adjustment of SO_2_, NO_2_ and CO. **Figure S1.** City-specific exposure–response curves for the association between O_3_ concentrations at lag0–1 and percentage change in the risk of pediatric pneumonia hospital admissions.

## Declarations

### Availability of data and materials

1. ChinaHighAirPollutants (CHAP) dataset: https://weijing-rs.github.io/product.html

2. Resource and Environmental Science Data Platform, the Institute of Geographic Sciences and Natural Resources Research of the Chinese Academy of Sciences: https://www.resdc.cn/

3. The FUTURE Database: available on request
