# Supplementary material for: Short-term effects of ambient ozone on pediatric pneumonia hospital admissions: a multi-city case-crossover study in China
Source: Environ Health Prev Med. 2025 Sep 23;30:75. doi: 10.1265/ehpm.25-00242 (PMC12483759; doi:10.1265/ehpm.25-00242)
Supplement: Supplementary file 1 — Additional file 1: Table S1. Demographic characteristics of the study population. Table S2. Summary distribution of O3 concentrations on case days across warm and cold seasons. Table S3. Spearman’s correlation coefficients among air pollutants and meteorological conditions on case days. Table S4. Pooled percentage change in the risk of pediatric pneumonia per 10 µg/m3 O3 increase. Table S5. City-specific percentage change in the risk of pediatric pneumonia hospital admissions per 10 µg/m3 increase in O3 exposure at different lag periods. Table S6. City-specific and pooled percentage change in the risk of pediatric pneumonia hospital admissions per 10 µg/m3 increase in O3 exposure at lag0–1 in stratified analyses. Table S7. City-specific and pooled percentage change in the risk of pediatric pneumonia hospital admissions per 10 µg/m3 increase in O3 exposure at lag0–1 in sensitivity analyses. Table S8. City-specific and pooled percentage change in the risk of pediatric pneumonia hospital admissions per 10 µg/m3 increase in O3 exposure at lag0–1 with additional adjustment of SO2, NO2 and CO. Figure S1. City-specific exposure–response curves for the association between O3 concentrations at lag0–1 and percentage change in the risk of pediatric pneumonia hospital admissions. [file ehpm-30-075-s001.docx]

**Supplementary materials**

**Table S1.** Demographic characteristics of the study population

**Table S2.** Summary distribution of O₃ concentrations on case days across warm and cold seasons

**Table S3.** Spearman’s correlation coefficients among air pollutants and meteorological conditions on case days

**Table S4.** Pooled percentage change in the risk of pediatric pneumonia per 10 μg/m^3^ O₃ increase

**Table S5.** City-specific percentage change in the risk of pediatric pneumonia hospital admissions per 10 μg/m³ increase in O₃ exposure at different lag periods

**Table S6.** City-specific and pooled percentage change in the risk of pediatric pneumonia hospital admissions per 10 μg/m³ increase in O₃ exposure at lag0–1 in stratified analyses

**Table S7.** City-specific and pooled percentage change in the risk of pediatric pneumonia hospital admissions per 10 μg/m³ increase in O₃ exposure at lag0–1 in sensitivity analyses

**Table S8.** City-specific and pooled percentage change in the risk of pediatric pneumonia hospital admissions per 10 μg/m³ increase in O₃ exposure at lag0–1 with additional adjustment of SO_2_, NO_2_ and CO

**Figure S1.** City-specific exposure–response curves for the association between O₃ concentrations at lag0–1 and percentage change in the risk of pediatric pneumonia hospital admissions

**Supplementary Table S1.** Demographic characteristics of the study population

| **City** | **Number** | **Sex** | | **Age** | | **Season at hospital admission** | |
| --- | --- | --- | --- | --- | --- | --- | --- |
|  | **of inpatients** | **Boys**  **No. (%)** | **Girls**  **No. (%)** | **≤3y**  **No. (%)** | **3-6y**  **No. (%)** | **Warm**  **No. (%)** | **Cold**  **No. (%)** |
| Dalian | 11217 | 6050 (53.9) | 5167 (46.1) | 4960 (44.2) | 6257 (55.8) | 4767 (42.5) | 6450 (57.5) |
| Kunming | 10624 | 6256 (58.9) | 4368 (41.1) | 8446 (79.5) | 2178 (20.5) | 4203 (39.6) | 6421 (60.4) |
| Nanchang | 17762 | 11551 (65) | 6211 (35) | 14486 (81.6) | 3276 (18.4) | 6034 (34) | 11728 (66) |
| Shijiazhuang | 25632 | 15446 (60.3) | 10186 (39.7) | 19199 (74.9) | 6433 (25.1) | 9822 (38.3) | 15810 (61.7) |
| Shenzhen | 20687 | 12414 (60) | 8273 (40) | 14685 (71) | 6002 (29) | 10900 (52.7) | 9787 (47.3) |
| Xi’an | 22500 | 13263 (58.9) | 9237 (41.1) | 14967 (66.5) | 7533 (33.5) | 8529 (37.9) | 13971 (62.1) |
| Zhengzhou | 29048 | 18358 (63.2) | 10690 (36.8) | 21413 (73.7) | 7635 (26.3) | 11103 (38.2) | 17945 (61.8) |
| Total | 137470 | 83338 (60.6) | 54132 (39.4) | 98156 (71.4) | 39314 (28.6) | 55358 (40.3) | 82112 (59.7) |

Warm season: from April to September; Cold season: October to March

**Supplementary Table S2.** Summary distribution of O₃ concentrations on case days across warm and cold seasons

| **City** | **Median（25th, 75th), μg/m^3^** | |
| --- | --- | --- |
|  | **Warm season** | **Cold season** |
| Dalian | 114.6 (100.7, 132.9) | 64.5 (56.7, 78.4) |
| Kunming | 90.6 (72.5, 116.6) | 82.0 (70.1, 94.3) |
| Nanchang | 108.6 (87.9, 129.6) | 69.2 (48.9, 93.0) |
| Shijiazhuang | 145.1 (118.6, 174.2) | 55.4 (39.3, 76.5) |
| Shenzhen | 81.1 (61.2, 120.4) | 92.1 (69.4, 123.4) |
| Xi’an | 118.2 (97.1, 135.1) | 61.0 (50.0, 77.9) |
| Zhengzhou | 149.5 (121.5, 180.0) | 61.6 (44.5, 85.7) |
| Overall | 119.7 (91.7, 148.1) | 66.3 (49.4, 88.5) |

Warm season: from April to September; Cold season: October to March

Abbreviations: 25th, the 25th percentile; 75th, the 75th percentile

**Supplementary Table S3.** Spearman’s correlation coefficients among air pollutants and meteorological conditions on case days

|  | **Temperature** | **Relative humidity** | **O_3_** | **PM_2.5_** |
| --- | --- | --- | --- | --- |
| **Dalian** |  |  |  |  |
| Temperature | 1.00 | 0.51^*^ | 0.73^*^ | -0.33^*^ |
| Relative humidity | 0.51^*^ | 1.00 | 0.24^*^ | 0.16^*^ |
| O_3_ | 0.73^*^ | 0.24^*^ | 1.00 | -0.16^*^ |
| PM_2.5_ | -0.33^*^ | 0.16^*^ | -0.16^*^ | 1.00 |
| **Kunming** |  |  |  |  |
| Temperature | 1.00 | 0.04^*^ | 0.25^*^ | -0.31^*^ |
| Relative humidity | 0.04^*^ | 1.00 | -0.77^*^ | -0.40^*^ |
| O_3_ | 0.25^*^ | -0.77^*^ | 1.00 | 0.45^*^ |
| PM_2.5_ | -0.31^*^ | -0.40^*^ | 0.45^*^ | 1.00 |
| **Nanchang** |  |  |  |  |
| Temperature | 1.00 | -0.14^*^ | 0.60^*^ | -0.37^*^ |
| Relative humidity | -0.14^*^ | 1.00 | -0.64^*^ | -0.30^*^ |
| O_3_ | 0.60^*^ | -0.64^*^ | 1.00 | -0.03^*^ |
| PM_2.5_ | -0.37^*^ | -0.30^*^ | -0.03^*^ | 1.00 |
| **Shijiazhuang** |  |  |  |  |
| Temperature | 1.00 | 0.22^*^ | 0.86^*^ | -0.49^*^ |
| Relative humidity | 0.22^*^ | 1.00 | -0.05^*^ | 0.31^*^ |
| O_3_ | 0.86^*^ | -0.05^*^ | 1.00 | -0.53^*^ |
| PM_2.5_ | -0.49^*^ | 0.31^*^ | -0.53^*^ | 1.00 |
| **Shenzhen** |  |  |  |  |
| Temperature | 1.00 | 0.17^*^ | 0.12^*^ | -0.45^*^ |
| Relative humidity | 0.17^*^ | 1.00 | -0.59^*^ | -0.48^*^ |
| O_3_ | 0.12^*^ | -0.59^*^ | 1.00 | 0.55^*^ |
| PM_2.5_ | -0.45^*^ | -0.48^*^ | 0.55^*^ | 1.00 |
| **Xi’an** |  |  |  |  |
| Temperature | 1.00 | 0.02^*^ | 0.74^*^ | -0.72^*^ |
| Relative humidity | 0.02^*^ | 1.00 | -0.37^*^ | -0.04^*^ |
| O_3_ | 0.74^*^ | -0.37^*^ | 1.00 | -0.56^*^ |
| PM_2.5_ | -0.72^*^ | -0.04^*^ | -0.56^*^ | 1.00 |
| **Zhengzhou** |  |  |  |  |
| Temperature | 1.00 | 0.10^*^ | 0.83^*^ | -0.61^*^ |
| Relative humidity | 0.10^*^ | 1.00 | -0.16^*^ | 0.17^*^ |
| O_3_ | 0.83^*^ | -0.16^*^ | 1.00 | -0.56^*^ |
| PM_2.5_ | -0.61^*^ | 0.17^*^ | -0.56^*^ | 1.00 |

^*^ *P*<0.05

**Supplementary Table S4.** Pooled percentage change in the risk of pediatric pneumonia per 10 μg/m^3^ O₃ increase

| Lag | Percentage Change | 95% CI | *P* value |
| --- | --- | --- | --- |
| lag0 | 1.07 | (0.09, 2.06) | **0.033** |
| lag1 | 0.96 | (0.56, 1.37) | **< 0.001** |
| lag2 | 0.15 | (-0.21, 0.50) | 0.414 |
| lag3 | -0.14 | (-0.48, 0.21) | 0.430 |
| lag4 | -0.27 | (-0.74, 0.21) | 0.271 |
| lag5 | -0.29 | (-0.85, 0.27) | 0.314 |
| lag6 | -0.13 | (-0.75, 0.49) | 0.684 |
| lag0–1 | 1.57 | (0.67, 2.48) | **< 0.001** |
| lag0–2 | 1.16 | (0.55, 1.78) | **< 0.001** |
| lag0–3 | 0.71 | (0.30, 1.12) | **< 0.001** |
| lag0–4 | 0.41 | (0.00, 0.83) | 0.051 |
| lag0–5 | 0.19 | (-0.34, 0.73) | 0.479 |
| lag0–6 | 0.11 | (-0.62, 0.84) | 0.770 |

Abbreviations: CI, Confidence Interval; lag1, the previous day; lag2, the previous 2 days; lag3, the previous 3 days; lag4, the previous 4 days; lag5, the previous 5 days; lag6, the previous 6 days; lag0–1, moving average of the case day and the previous day; lag0–2, moving average of the case day and the previous 2 days; lag0–3, moving average of the case day and the previous 3 days; lag0–4, moving average of the case day and the previous 4 days; lag0–5, moving average of the case day and the previous 5 days; lag0–6, moving averages of the case day and 1 to 6 preceding days.

**Supplementary Table S5.** City-specific percentage change in the risk of pediatric pneumonia hospital admissions per 10 μg/m³ increase in O₃ exposure at different lag periods

| **City** | **Percentage Change** | **95% CI** | ***P* value** |
| --- | --- | --- | --- |
| **Dalian** |  |  |  |
| lag0 | 0.55 | (-1.07, 2.19) | 0.508 |
| lag1 | 0.96 | (-0.46, 2.39) | 0.187 |
| lag2 | 0.78 | (-0.54, 2.12) | 0.249 |
| lag3 | 0.94 | (-0.36, 2.26) | 0.156 |
| lag4 | 0.87 | (-0.40, 2.15) | 0.180 |
| lag5 | 1.55 | (0.28, 2.83) | **0.016** |
| lag6 | 1.93 | (0.67, 3.20) | **0.003** |
| lag0-1 | 1.14 | (-0.68, 2.99) | 0.223 |
| lag0-2 | 1.41 | (-0.53, 3.38) | 0.155 |
| lag0-3 | 1.77 | (-0.28, 3.87) | 0.092 |
| lag0-4 | 2.08 | (-0.09, 4.30) | 0.060 |
| lag0-5 | 2.79 | (0.48, 5.15) | **0.017** |
| lag0-6 | 3.71 | (1.26, 6.23) | **0.003** |
| **Kunming** |  |  |  |
| lag0 | 0.18 | (-2.55, 2.98) | 0.901 |
| lag1 | 0.24 | (-1.93, 2.45) | 0.832 |
| lag2 | 0.37 | (-1.39, 2.16) | 0.683 |
| lag3 | 0.27 | (-1.33, 1.89) | 0.746 |
| lag4 | 0.16 | (-1.36, 1.71) | 0.837 |
| lag5 | -0.28 | (-1.75, 1.22) | 0.715 |
| lag6 | 0.19 | (-2.20, 2.64) | 0.876 |
| lag0-1 | 0.30 | (-2.56, 3.25) | 0.837 |
| lag0-2 | 0.47 | (-2.23, 3.24) | 0.735 |
| lag0-3 | 0.49 | (-2.07, 3.12) | 0.710 |
| lag0-4 | 0.45 | (-2.03, 3.00) | 0.723 |
| lag0-5 | 0.24 | (-2.18, 2.71) | 0.851 |
| lag0-6 | -0.01 | (-1.47, 1.47) | 0.986 |
| **Nanchang** |  |  |  |
| lag0 | 0.57 | (-0.43, 1.58) | 0.262 |
| lag1 | 1.48 | (0.61, 2.35) | **<0.001** |
| lag2 | 0.49 | (-0.23, 1.21) | 0.180 |
| lag3 | -0.18 | (-0.85, 0.49) | 0.591 |
| lag4 | -0.74 | (-1.39, -0.09) | **0.026** |
| lag5 | -0.47 | (-1.10, 0.17) | 0.148 |
| lag6 | -0.41 | (-1.03, 0.21) | 0.197 |
| lag0-1 | 1.70 | (0.54, 2.87) | **0.004** |
| lag0-2 | 1.53 | (0.38, 2.69) | **0.009** |
| lag0-3 | 0.99 | (-0.14, 2.13) | 0.086 |
| lag0-4 | 0.35 | (-0.77, 1.49) | 0.541 |
| lag0-5 | 0.05 | (-1.09, 1.19) | 0.935 |
| lag0-6 | -0.16 | (-1.31, 1.00) | 0.786 |
| **Shijiazhuang** |  |  |  |
| lag0 | 2.67 | (1.80, 3.56) | **<0.001** |
| lag1 | 1.66 | (0.92, 2.41) | **<0.001** |
| lag2 | -0.41 | (-1.05, 0.24) | 0.218 |
| lag3 | -0.58 | (-1.19, 0.04) | 0.066 |
| lag4 | -1.16 | (-1.76, -0.55) | **<0.001** |
| lag5 | -0.97 | (-1.55, -0.38) | **0.001** |
| lag6 | -0.90 | (-1.48, -0.32) | **0.002** |
| lag0-1 | 3.16 | (2.17, 4.16) | **<0.001** |
| lag0-2 | 1.85 | (0.85, 2.86) | **<0.001** |
| lag0-3 | 1.05 | (0.04, 2.06) | **0.042** |
| lag0-4 | 0.21 | (-0.81, 1.25) | 0.685 |
| lag0-5 | -0.32 | (-1.36, 0.73) | 0.551 |
| lag0-6 | -0.71 | (-1.76, 0.35) | 0.190 |
| **Shenzhen** |  |  |  |
| lag0 | 0.39 | (-0.29, 1.07) | 0.264 |
| lag1 | 0.57 | (0.01, 1.14) | **0.047** |
| lag2 | 0.43 | (-0.05, 0.92) | 0.077 |
| lag3 | 0.04 | (-0.41, 0.50) | 0.855 |
| lag4 | 0.25 | (-0.19, 0.70) | 0.267 |
| lag5 | 0.30 | (-0.14, 0.74) | 0.183 |
| lag6 | 0.22 | (-0.21, 0.66) | 0.320 |
| lag0-1 | 0.71 | (-0.02, 1.46) | 0.058 |
| lag0-2 | 0.80 | (0.07, 1.54) | **0.032** |
| lag0-3 | 0.63 | (-0.10, 1.36) | 0.092 |
| lag0-4 | 0.65 | (-0.09, 1.39) | 0.084 |
| lag0-5 | 0.70 | (-0.05, 1.46) | 0.066 |
| lag0-6 | 0.73 | (-0.04, 1.50) | 0.063 |
| **Xi’an** |  |  |  |
| lag0 | 3.03 | (1.74, 4.35) | **<0.001** |
| lag1 | 0.70 | (-0.40, 1.82) | 0.214 |
| lag2 | 0.17 | (-0.76, 1.11) | 0.722 |
| lag3 | -0.96 | (-1.83, -0.08) | **0.033** |
| lag4 | -0.17 | (-1.02, 0.69) | 0.702 |
| lag5 | -0.75 | (-1.58, 0.09) | 0.080 |
| lag6 | -0.41 | (-1.24, 0.43) | 0.339 |
| lag0-1 | 3.10 | (1.48, 4.74) | **<0.001** |
| lag0-2 | 2.31 | (0.68, 3.97) | **0.005** |
| lag0-3 | 0.91 | (-0.70, 2.55) | 0.268 |
| lag0-4 | 0.62 | (-1.00, 2.27) | 0.453 |
| lag0-5 | 0.05 | (-1.59, 1.72) | 0.950 |
| lag0-6 | -0.19 | (-1.86, 1.51) | 0.825 |
| **Zhengzhou** |  |  |  |
| lag0 | -0.17 | (-0.90, 0.56) | 0.645 |
| lag1 | 0.70 | (0.09, 1.31) | **0.025** |
| lag2 | -0.18 | (-0.71, 0.35) | 0.504 |
| lag3 | 0.10 | (-0.41, 0.60) | 0.712 |
| lag4 | -0.35 | (-0.84, 0.14) | 0.160 |
| lag5 | -0.76 | (-1.24, -0.28) | **0.002** |
| lag6 | -0.62 | (-1.10, -0.13) | **0.012** |
| lag0-1 | 0.53 | (-0.29, 1.35) | 0.207 |
| lag0-2 | 0.21 | (-0.61, 1.04) | 0.614 |
| lag0-3 | 0.22 | (-0.60, 1.06) | 0.597 |
| lag0-4 | -0.02 | (-0.85, 0.81) | 0.960 |
| lag0-5 | -0.40 | (-1.23, 0.44) | 0.348 |
| lag0-6 | -0.63 | (-1.47, 0.22) | 0.148 |

Abbreviations: CI, Confidence Interval; lag0, the case day; lag1, the previous day; lag2, the previous two days; lag3, the previous three days; lag4, the previous four days; lag5, the previous five days; lag6, the previous six days; lag0-1, moving average of the case day and the previous day; lag0-2, moving average of the case day and the previous two days; lag0-3, moving average of the case day and the previous three days; lag0-4, moving average of the case day and the previous four days; lag0-5, moving average of the case day and the previous five days; lag0-6, moving average of the case day and the previous six days

**Supplementary Table S6.** City-specific and pooled percentage change in the risk of pediatric pneumonia hospital admissions per 10 μg/m³ increase in O₃ exposure at lag0–1 in stratified analyses

| **Subgroup** | **Percentage Change** | **95% CI** | ***P* value** |
| --- | --- | --- | --- |
| **Gender** |  |  |  |
| Male |  |  |  |
| Dalian | 1.59 | (-0.91, 4.20) | 0.214 |
| Kunming | -1.61 | (-5.25, 2.17) | 0.399 |
| Nanchang | 2.02 | (0.58, 3.49) | **0.006** |
| Shijiazhuang | 3.00 | (1.73, 4.30) | **<0.001** |
| Shenzhen | 1.10 | (0.14, 2.07) | **0.024** |
| Xi’an | 2.80 | (0.69, 5.00) | **0.009** |
| Zhengzhou | 0.31 | (-0.73, 1.37) | 0.558 |
| Pooled estimate | 1.53 | (0.61, 2.46) | **0.001** |
| Female |  |  |  |
| Dalian | 0.65 | (-1.99, 3.36) | 0.634 |
| Kunming | 3.17 | (-1.39, 7.95) | 0.176 |
| Nanchang | 1.12 | (-0.80, 3.10) | 0.255 |
| Shijiazhuang | 3.42 | (1.84, 5.02) | **<0.001** |
| Shenzhen | 0.14 | (-1.01, 1.30) | 0.816 |
| Xi’an | 3.51 | (1.01, 6.10) | **0.006** |
| Zhengzhou | 0.84 | (-0.48, 2.18) | 0.213 |
| Pooled estimate | 1.59 | (0.45, 2.75) | **0.006** |
| **Age group** |  |  |  |
| ≤3y |  |  |  |
| Dalian | 2.50 | (-0.31, 5.40) | 0.082 |
| Kunming | -0.83 | (-4.01, 2.44) | 0.613 |
| Nanchang | 1.45 | (0.17, 2.75) | **0.027** |
| Shijiazhuang | 2.75 | (1.59, 3.92) | **<0.001** |
| Shenzhen | 0.69 | (-0.20, 1.57) | 0.127 |
| Xi’an | 3.16 | (1.16, 5.20) | **0.002** |
| Zhengzhou | 0.40 | (-0.57, 1.38) | 0.419 |
| Pooled estimate | 1.46 | (0.54, 2.39) | **0.002** |
| 3-6y |  |  |  |
| Dalian | 0.16 | (-2.22, 2.59) | 0.900 |
| Kunming | 4.83 | (-1.65, 11.70) | 0.148 |
| Nanchang | 2.71 | (0.06, 5.40) | **0.045** |
| Shijiazhuang | 4.30 | (2.38, 6.30) | **<0.001** |
| Shenzhen | 0.80 | (-0.55, 2.20) | 0.247 |
| Xi’an | 3.05 | (0.34, 5.80) | **0.027** |
| Zhengzhou | 0.83 | (-0.69, 2.38) | 0.285 |
| Pooled estimate | 1.97 | (0.68, 3.27) | **0.003** |
| **Admission season** |  |  |  |
| Warm season |  |  |  |
| Dalian | 0.18 | (-2.25, 2.70) | 0.889 |
| Kunming | 0.45 | (-3.38, 4.43) | 0.822 |
| Nanchang | 1.27 | (-0.64, 3.22) | 0.193 |
| Shijiazhuang | 4.32 | (2.84, 5.82) | **<0.001** |
| Shenzhen | 0.94 | (-0.15, 2.00) | 0.092 |
| Xi’an | 5.74 | (3.35, 8.17) | **<0.001** |
| Zhengzhou | 0.47 | (-0.83, 1.80) | 0.479 |
| Pooled estimate | 1.95 | (0.36, 3.56) | **0.016** |
| Cold season |  |  |  |
| Dalian | 0.71 | (-2.41, 3.93) | 0.659 |
| Kunming | -0.66 | (-4.95, 3.83) | 0.770 |
| Nanchang | 1.58 | (0.10, 3.08) | **0.037** |
| Shijiazhuang | 0.96 | (-0.58, 2.50) | 0.223 |
| Shenzhen | 0.84 | (-0.24, 1.95) | 0.129 |
| Xi’an | 1.04 | (-1.28, 3.40) | 0.383 |
| Zhengzhou | 1.45 | (0.29, 2.63) | **0.014** |
| Pooled estimate | 1.12 | (0.52, 1.72) | **<0.001** |

Abbreviations: CI, Confidence Interval; lag0-1, moving average of the case day and the previous day

**Supplementary Table S7.** City-specific and pooled percentage change in the risk of pediatric pneumonia hospital admissions per 10 μg/m³ increase in O₃ exposure at lag0–1 in sensitivity analyses

| **Model** | **Percentage Change** | **95% CI** | ***P* value** |
| --- | --- | --- | --- |
| **Model 1** |  |  |  |
| Dalian | 1.00 | (-0.78, 2.81) | 0.273 |
| Kunming | 0.54 | (-2.31, 3.48) | 0.712 |
| Nanchang | 1.66 | (0.55, 2.78) | **0.003** |
| Shijiazhuang | 2.93 | (1.94, 3.92) | **<0.001** |
| Shenzhen | 0.79 | (0.07, 1.53) | **0.032** |
| Xi’an | 2.84 | (1.26, 4.45) | **<0.001** |
| Zhengzhou | 0.61 | (-0.20, 1.44) | 0.142 |
| Pooled estimate | 1.52 | (0.73, 2.32) | **<0.001** |
| **Model 2** |  |  |  |
| Dalian | 1.13 | (-0.71, 3.00) | 0.232 |
| Kunming | 0.21 | (-2.66, 3.16) | 0.887 |
| Nanchang | 1.76 | (0.60, 2.95) | **0.003** |
| Shijiazhuang | 2.99 | (1.99, 3.99) | **<0.001** |
| Shenzhen | 0.72 | (-0.02, 1.47) | 0.056 |
| Xi’an | 3.29 | (1.66, 4.96) | **<0.001** |
| Zhengzhou | 0.58 | (-0.24, 1.41) | 0.167 |
| Pooled estimate | 1.58 | (0.69, 2.47) | **<0.001** |
| **Model 3** |  |  |  |
| Dalian | 0.64 | (-1.74, 3.08) | 0.600 |
| Kunming | 3.37 | (0.66, 6.16) | **0.015** |
| Nanchang | 0.82 | (-0.38, 2.03) | 0.183 |
| Shijiazhuang | 1.93 | (-0.29, 4.20) | 0.089 |
| Shenzhen | 1.97 | (1.22, 2.73) | **<0.001** |
| Xi’an | 0.68 | (-0.77, 2.14) | 0.361 |
| Zhengzhou | 1.35 | (0.78, 1.94) | **<0.001** |
| Pooled estimate | 1.45 | (1.01, 1.90) | **<0.001** |

Model 1: metrological conditions with 2 degree of freedom in natural cubic spline function

Model 2: metrological conditions with 4 degree of freedom in natural cubic spline function

Model 3: subjects matched with raster data of air pollutants and nearest monitoring station data of meteorological conditions (n=64525)

Abbreviations: CI, Confidence Interval; lag0-1, moving average of the case day and the previous day

**Supplementary Table S8.** City-specific and pooled percentage change in the risk of pediatric pneumonia hospital admissions per 10 μg/m³ increase in O₃ exposure at lag0–1 with additional adjustment of SO_2_, NO_2_ and CO

| **Model** | **Percentage Change** | **95% CI** | ***P* value** | ***P* for interaction** |
| --- | --- | --- | --- | --- |
| **Main model** | 1.57 | (0.67, 2.48) | **<0.001** | reference |
| **+SO_2_** |  |  |  | 0.398 |
| Dalian | 1.31 | (-0.53, 3.18) | 0.164 |  |
| Kunming | 0.43 | (-2.50, 3.46) | 0.775 |  |
| Nanchang | 1.70 | (0.54, 2.87) | **0.004** |  |
| Shijiazhuang | 3.17 | (2.18, 4.18) | **<0.001** |  |
| Shenzhen | 0.72 | (-0.02, 1.50) | 0.056 |  |
| Xi’an | 3.11 | (1.49, 4.75) | **<0.001** |  |
| Zhengzhou | 0.50 | (-0.32, 1.33) | 0.231 |  |
| Pooled estimate | 1.60 | (0.70, 2.51) | **<0.001** |  |
| **+NO_2_** |  |  |  | 0.397 |
| Dalian | 1.50 | (-0.38, 3.41) | 0.118 |  |
| Kunming | -0.31 | (-3.26, 2.74) | 0.841 |  |
| Nanchang | 1.68 | (0.52, 2.85) | **0.004** |  |
| Shijiazhuang | 3.59 | (2.57, 4.62) | **<0.001** |  |
| Shenzhen | 0.72 | (-0.02, 1.46) | 0.057 |  |
| Xi’an | 3.08 | (1.45, 4.73) | **<0.001** |  |
| Zhengzhou | 0.50 | (-0.32, 1.33) | 0.234 |  |
| Pooled estimate | 1.63 | (0.62, 2.65) | **0.002** |  |
| **+CO** |  |  |  | 0.397 |
| Dalian | 1.23 | (-0.60, 3.10) | 0.189 |  |
| Kunming | 0.06 | (-2.82, 3.04) | 0.966 |  |
| Nanchang | 1.79 | (0.62, 2.96) | **0.002** |  |
| Shijiazhuang | 3.53 | (2.52, 4.54) | **<0.001** |  |
| Shenzhen | 0.71 | (-0.02, 1.46) | 0.058 |  |
| Xi’an | 3.12 | (1.51, 4.77) | **<0.001** |  |
| Zhengzhou | 0.51 | (-0.31, 1.35) | 0.224 |  |
| Pooled estimate | 1.64 | (0.65, 2.64) | **0.001** |  |

Abbreviations: CI, Confidence Interval; lag0-1, moving average of the case day and the previous day

**Figure S1.** City-specific exposure–response curves for the association between O₃ concentrations at lag0–1 and percentage change in the risk of pediatric pneumonia hospital admissions


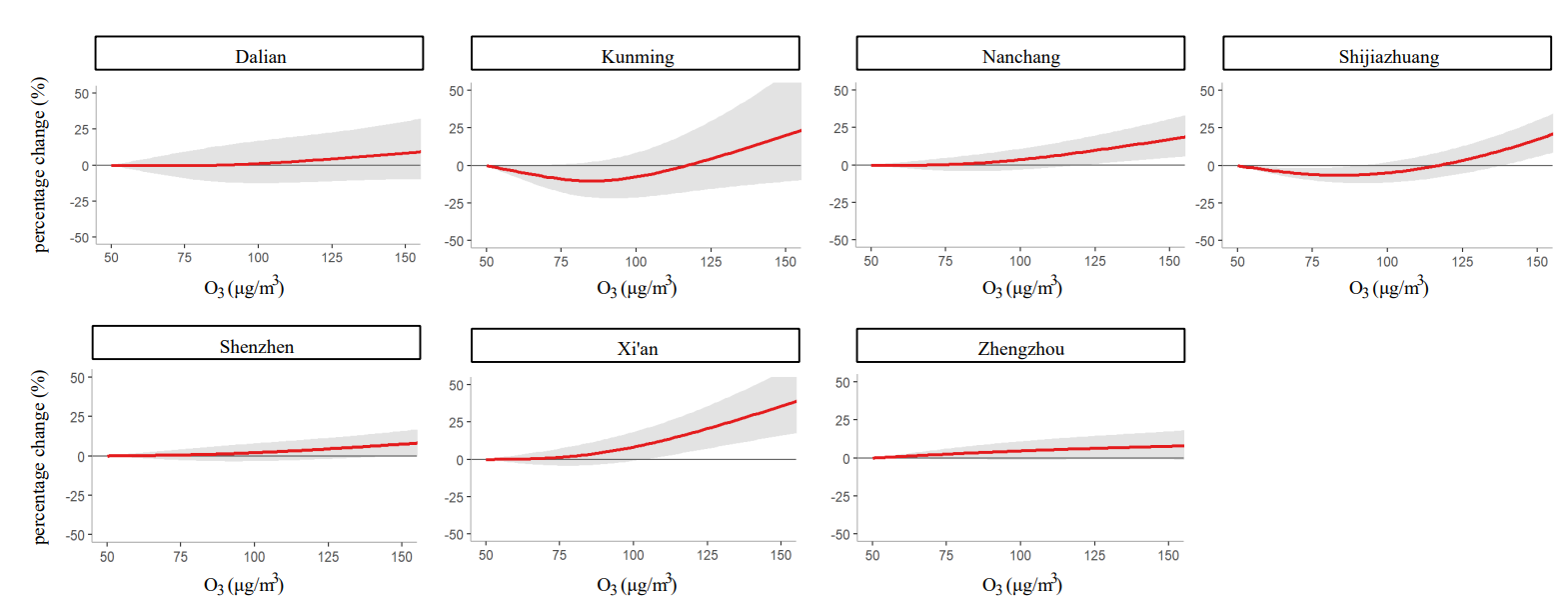


Abbreviations: lag0-1, moving average of the case day and the previous day
